# Phenolic Compounds from *Pyrus communis* Residues: Mechanisms of Antibacterial Action and Therapeutic Applications

**DOI:** 10.3390/antibiotics14030280

**Published:** 2025-03-08

**Authors:** Jessica Ribeiro, Vanessa Silva, Gilberto Igrejas, Lillian Barros, Sandrina A. Heleno, Filipa S. Reis, Patrícia Poeta

**Affiliations:** 1 Microbiology and Antibiotic Resistance Team (MicroART), University of Trás-os-Montes and Alto Douro (UTAD), 5000-801 Vila Real, Portugal; jessicalribeiro97@gmail.com (J.R.); vanessasilva@utad.pt (V.S.); 2 Associated Laboratory for Green Chemistry (LAQV-REQUIMTE), University NOVA of Lisbon, 2829-516 Lisbon, Portugal; 3 Centro de Investigação de Montanha (CIMO), La SusTEC, Instituto Politécnico de Bragança (IPB), 5300-253 Bragança, Portugal; lillian@ipb.pt (L.B.); sheleno@ipb.pt (S.A.H.); freis@ipb.pt (F.S.R.); 4 Department of Genetics and Biotechnology, University of Trás-os-Montes and Alto Douro (UTAD), 5000-801 Vila Real, Portugal; gigrejas@utad.pt; 5 Functional Genomics and Proteomics Unit, University of Trás-os-Montes and Alto Douro (UTAD), 5000-801 Vila Real, Portugal; 6 Associate Laboratory for Animal and Veterinary Science (AL4AnimalS), University of Trás-os-Montes and Alto Douro (UTAD), 5000-801 Vila Real, Portugal; 7 Veterinary and Animal Research Centre (CECAV), University of Trás-os-Montes and Alto Douro (UTAD), 5000-801 Vila Real, Portugal

**Keywords:** antioxidant properties, chlorogenic acid, hydroquinone, bacterial resistance, natural therapeutics, oxidative stress, plant-derived compounds, secondary metabolites

## Abstract

Background/Objectives: The food industry produces substantial amounts of fruit byproducts, which are often discarded despite their high content of bioactive compounds with potential therapeutic applications. *Pyrus communis* (pear) residues, which are particularly rich in phenolic compounds, represent a valuable yet underutilized resource. These byproducts have demonstrated significant antioxidant and antibacterial properties, suggesting their potential for medical and pharmaceutical applications. This review aims to provide a comprehensive analysis of the phenolic profile of *P. communis* byproducts, emphasizing their antioxidant and antibacterial mechanisms and their prospective use in combating oxidative stress and antibacterial resistance. Methods: A comprehensive review of the key phenolic compounds from *P. communis* residues was conducted using ScienceDirect and Google Scholar databases (from 2014 to 2024). Studies assessing antioxidant and antibacterial activities were reviewed, with a focus on their mechanisms of action against Gram-positive and Gram-negative bacterial pathogens. Results: A minimum of 14 distinct phenolic compounds were identified among *P. communis* residues. However, chlorogenic acid and catechin were identified as the primary contributors to the antioxidant activity of *P. communis* residues. Hydroquinone and chlorogenic acid exhibited strong antibacterial effects through membrane disruption, enzyme inhibition, and metabolic interference. Despite this potential, hydroquinone’s cytotoxicity and regulatory concerns limit its direct pharmaceutical application. Conclusions: While *P. communis* phenolics show promise as natural antibacterial agents, future research should address bioavailability, extraction standardization, and safe formulation strategies. Investigating their synergy with conventional antibiotics and improving stability for cosmetic applications are key steps toward their practical use. In vivo and clinical studies are crucial to validating their therapeutic potential and ensuring regulatory approval.

## 1. Introduction

The agro-industrial sector generates substantial amounts of food waste, much of which remains underutilized despite its potential bioactive properties [1]. *Pyrus communis* (pear) is one of the most widely cultivated fruits worldwide, particularly in Asia, Africa, and Europe [2,3]. In Europe, cultivars such as Conference, Williams BC, and Rocha dominate pear production [4]. Among these, Pêra Rocha stands out as an exclusive Portuguese variety, recognized under the Protected Designation of Origin (PDO) label for its distinctive sensory attributes and economic significance [5]. In 2023, Portugal produced approximately 138,000 tons of pears, making it the country’s second most-produced fruit [4]. However, the processing of these pears generates substantial residues, including peels, seeds, and pomace, which are often discarded despite their rich bioactive composition and potential applications in food and health industries.

Food waste not only poses an economic and environmental burden but also a missed opportunity for sustainable valorization. The fruit juice industry alone discards between 20% and 60% of raw material as waste, much of which contains bioactive molecules with potential health applications [6]. In Portugal, an estimated 1.93 million tons of food was wasted in 2022, further highlighting the urgency of developing innovative strategies to repurpose agro-industrial residues [7]. Recent research has emphasized the potential of phenolic-rich byproducts in food preservation, pharmaceuticals, and antibacterial applications in food preservation, pharmaceuticals, and antibacterial treatments, offering a promising alternative to synthetic additives and conventional antibiotics [8,9,10]. Phenolic compounds, secondary metabolites found in plants, play a key role in preventing oxidative stress by scavenging free radicals and inhibiting lipid peroxidation, which contributes to their use in functional foods and pharmaceuticals [11]. These compounds have also shown promising antibacterial properties by disrupting bacterial membranes, inhibiting essential enzymes, and interfering with microbial metabolism [12,13]. Given the alarming rise in antibacterial resistance, there is an increasing need for natural compounds that are capable of combating multidrug-resistant pathogens [14].

Despite extensive research on the antioxidant properties of phenolic compounds, their specific antibacterial mechanisms—particularly their effects on resistant bacterial strains—remain underexplored [15,16]. While some studies have demonstrated the antibacterial potential of *P. communis* extracts, the exact interactions between these bioactive compounds and bacterial cells are not yet fully understood [17,18,19]. Investigating these mechanisms is crucial for optimizing their application in infection control, food preservation, and pharmaceutical formulations. Furthermore, there is limited information on the bioavailability and metabolic stability of phenolic compounds from pear residues, which are critical factors for their potential therapeutic applications. Additionally, the valorization of agro-industrial byproducts as natural antibacterial agents aligns with global efforts to promote sustainability and reduce food waste [20,21].

This review provides a comprehensive analysis of the phenolic composition of *P. communis* residues, with a focus on their antioxidant and antibacterial properties. Special attention is given to their effects on bacterial pathogens, their mechanisms of action, and their potential applications in addressing antibacterial resistance. By consolidating current knowledge and identifying research gaps, this work contributes to the development of sustainable and effective bioactive agents derived from agro-industrial byproducts. Furthermore, this review highlights future research directions, including the need for in vivo studies, the optimization of extraction methods, and the evaluation of synergistic effects with conventional antimicrobials.

## 2. Methodology of Research

A comprehensive literature review was conducted using ScienceDirect (https://www.sciencedirect.com/ accessed from January to April 2024) and Google Scholar (https://scholar.google.pt/ accessed from January to April 2024)) to identify relevant studies published between 2014 and 2024. The search terms included ‘*Pyrus communis*’, ‘phenolic compounds’, ‘antioxidant activity’, and ‘antibacterial activity’, which were combined. The review primarily includes peer-reviewed articles, with additional references from books and authoritative reports where relevant. Conference abstracts and non-English publications were excluded. Additional references were identified by examining the reference lists of selected articles.

## 3. Residues of *P. communis*

Pears are climacteric fruits that produce ethylene, accelerating ripening and influencing their color, texture, and nutritional attributes. The rapid maturation caused by ethylene can result in physiological disorders, senescence, and heightened vulnerability to pathogens, ultimately decreasing the fruit’s shelf-life [22]. Temperature also plays a critical role in this process [23]. To meet the standards of post-harvest handling and distribution, pears are frequently harvested before reaching full maturity [21]. Nevertheless, inadequate fruit quality and logistical problems are two leading causes of the yearly waste of numerous pears [24]. Post-harvest challenges, including physical damage, moisture loss, and disease, impact the appearance and storability of pears [21,25,26]. Nonetheless, fruits that have external defects and low internal values or were harvested at an incorrect maturity level may yet contain significant quantities of sugars, minerals, amino acids, and phenolic compounds, which contribute to their nutritional worth [20,24].

When pears reach the market, a significant portion is either unused or left unconsumed. Note that the pear fruit peduncle is inedible, whereas the core is edible; however, because of its cartilaginous nature and low digestibility, it is typically discarded alongside the seeds. In addition, the peduncle, peel, and core are biological residues that result from pear juice processing [27]. Research indicates up to 60% of processed fruit biomass is discarded without being utilized [28]. However, the extraction of phenolic compounds from agricultural waste can significantly reduce this impact and provide natural ingredients for the food and pharmaceutical industries to replace synthetic preservatives such as butylated hydroxytoluene (BHT) and parabens [9,10]. It is imperative to explore strategies that not only enhance economic efficiency but also contribute to environmental sustainability. A promising approach entails the valorization of discarded fruit through the production of value-added goods, encompassing fresh juices, tinned jellies and jams, alcoholic beverages, and dried fruits. These products serve not only as sources of biologically active compounds but also as a reservoir for diverse applications, including food additives, nutraceuticals, and cosmetic ingredients.

### Extraction Techniques and Phenolic Profile of P. communis Residues

Although there are numerous studies focused on the phenolic profile of *P. communis*, comparing its phenolic content among published data is difficult because of the differences in analytical methods, limited data on seeds and leaves, and significant fluctuations in component concentrations with respect to pear variety and the stage of maturity [29]. Table 1 summarizes the extraction conditions used in studies on the phenolic content of *P. communis* varieties and their respective yields, allowing for a comparison to determine the most effective method.

Among the studies analyzed, methanol-based extractions consistently yielded higher phenolic contents compared to ethanol-based methods. For instance, methanol with 1% ascorbic acid extracted up to 532.7 g GAE kg^−1^ from leaves in just 20 min, while methanol with 0.1% hydrochloric acid provided 116.3–173.1 g GAE kg^−1^ from peel and 85.3–146.4 g GAE kg^−1^ from pulp in 1 h [30,33]. In contrast, extractions using 70% ethanol for 12 h resulted in significantly lower yields (0.2–0.3 g GAE kg^−1^), suggesting that methanol, particularly when acidified, is a more effective solvent for phenolic recovery [35].

To optimize and standardize phenolic extraction, we propose a protocol using 100 mL methanol with 0.1% HCl, 10 g of sample, and a one-hour extraction time, which has proven to be both efficient and reproducible. For leaves, a modified approach with 5 mL methanol containing 1% ascorbic acid and a 20 min extraction time may be preferable, given its exceptional performance. These conditions ensure high phenolic yields while maintaining a practical extraction duration, making them suitable for broader applications in food, pharmaceutical, and nutraceutical research.

Overall, many studies have frequently identified chlorogenic acid and arbutin in various anatomical elements of the *P. communis* pear (Table 2) [15,29,30,31,32,33,34,35].

Studies consistently identify chlorogenic acid and arbutin as predominant phenolic compounds in *P*. *communis*. Chlorogenic acid is abundant in the peel, pulp, and core, while arbutin is prominent in the peduncle [32,34]. In contrast, Kolniak-Ostek [30] reported that the phenolic compound concentration was the highest in the leaves of *P. communis*. The peel, pulp, and seeds contained flavanols as the main phenolics, whereas flavonols were predominant in the leaves. The leaves had the highest concentration of phenolic acids, whereas the lowest concentration was detected in the pulp. Leaves also presented the main quantity of flavones, followed by peel and pulp. Flavones were absent in pear seeds, whereas the lowest level of arbutin was observed in the pulp. Anthocyanins were found solely in the peel [30]. In addition, Kolniak-Ostek and Oszmiański [29] discovered that phenolic compounds were more abundant in leaves, seeds, peel, and pulp; however, phenolic acids and their derivatives were identified as the primary phenolics found in pear leaves.

In the work by Li et al. [31], arbutin emerged as the dominant compound in the peel and pulp of various pear cultivars. In addition to arbutin, researchers have identified three main flavonoids (catechin, epicatechin, and quercetin), five phenolic acids (chlorogenic, gallic, ferulic, vanillic, and *p*-coumaric acids), and two triterpenes (oleanolic and ursolic acids) [31]. Akagić et al. [15] identified six phenolics in the peel of pears, which can be organized into four key phenolic classes: hydroxycinnamic acids (chlorogenic acid), glycosylated hydroquinone (arbutin), flavanols (epicatechin and catechin), and flavonols (quercetin 3-*O*-glucoside and quercetin 3-*O*-rutinoside). Flavonols exist in a glycosylated form, where sugar molecules are attached to the aglycone (the non-sugar portion), influencing their bioavailability and antioxidant activity [36]. These findings add to our knowledge of the chemical structure of pear peels. They indicate that the analyzed pear varieties contained glycosylated hydroquinone and hydroxycinnamic acid as their principal phenolic compounds, followed by flavanols and flavonols, in accordance with the findings described by Cui et al. [37].

In comparison to other metabolites, phenolic compounds present several advantages due to their distinctive and multifaceted beneficial properties. These compounds have revealed enhanced pharmacological and nutritional characteristics, including antibacterial [12], antioxidant, anti-inflammatory [13], and anti-cancer properties [38]. They function as antioxidants by scavenging free radicals, upregulating endogenous antioxidant pathways, and preventing oxidative stress-related damage [39]. The potential of phenolic compounds for modulating cellular components, regulating glucose metabolism, inhibiting cell proliferation, and enhancing intrinsic defense systems makes them attractive options for the improvement of human health and the treatment of diverse ailments [40].

## 4. Antioxidant Activity of Phenolic Compounds from *P. communis*

It is important to note that there are over 3000 cultivars of the pear tree, which is a cultivated plant that is widely distributed throughout Europe [41]. In 2023, the most produced pear varieties at the European level were Conference, followed by William BC and Rocha. Other cultivars, including Coscia-Ercollini, Abate Fetel, Doyenne du Comice, Guyot, Blanquilla, Kaiser, Passacrassana, and Durondeau, are similarly cultivated, although to a lesser extent [4]. The antioxidant properties and phenolic composition of three key pear varieties—Conference, Williams, and Rocha—have been explored extensively (Table 3), highlighting differences in their nutritional and functional qualities.

Studies consistently show that Conference pears exhibit lower antioxidant capacity compared to other cultivars. Frontini et al. [42] and Kolniak-Ostek et al. [44] attributed this to the decline in phenolic compounds during ripening and storage. Similarly, Ruiz-Torralba et al. [45] confirmed that Conference pears ranked among fruits with the lowest antioxidant activity when analyzed with or without the peel, though the antioxidant capacity was higher when the peel was intact. Chlorogenic acid, a major phenolic compound, was identified as a key determinant of antioxidant potential. Conversely, Liaudanskas et al. [46] and Commisso et al. [43] found that Conference pears usually contain lower levels of this compound, correlating with their reduced antioxidant capacity.

William’s pears demonstrated more variable results. While Kirca et al. [3] highlighted their relatively high antioxidant activity, particularly due to elevated catechin contents, other studies such as Akšić et al. [48] and Commisso et al. [43] reported lower antioxidant capacities in William’s pears, attributed to the reduced levels of key phenolics like chlorogenic acid. Notably, Magri et al. [47] demonstrated that applying an active edible coating to fresh-cut William’s pears improved their antioxidant activity by preserving bioactive compounds and reducing oxidative damage.

The Rocha pear, a prominent Portuguese cultivar, has been extensively studied for its phenolic content and antioxidant properties. Rocha pears are particularly rich in phenolic compounds, with chlorogenic acid dominating. Studies such as Fernandes et al. [51] and Lomba-Viana et al. [6] revealed that Rocha pear pomace, a byproduct of juice production, retains high antioxidant capacity, emphasizing its potential for nutraceutical applications. Drying processes significantly influence Rocha pears’ antioxidant properties, as shown by Santos et al. [49] and Onal et al. [50]. While drying reduces the overall antioxidant activity, ultrasonic pretreatment combined with higher drying temperatures (60 °C) was found to effectively preserve phenolic compounds and antioxidant capacity, demonstrating an efficient method for maintaining their nutritional value.

These findings underscore the importance of phenolic compositions in determining the antioxidant capacity of pears and emphasize the cultivar-specific effects of pre-treatments, processing methods, and storage on their nutritional qualities.

### Molecular Mechanisms of Antioxidant Action

Determining the mechanisms of action of phenolic compounds in several physiological processes, including cellular signal transduction, cell proliferation and differentiation, apoptosis, and inflammation, may provide significant insights into their pharmaceutical applications. The relationship between polyphenols and antioxidant capacity is complex and can be modulated by molecular structure and experimental conditions [44]. The antioxidant capacity of phenolics is predominantly influenced by the number and configuration of H-donating hydroxyl groups [52].

Chlorogenic acid, an active plant-derived antioxidant, has a unique molecular structure that facilitates its antioxidant properties. Its molecular structure includes five active hydroxyl groups at positions 3, 4, and 5 of the benzyl ring and positions 3′ and 4′ of the cinnamoyl moiety, along with one carboxyl group at position 1, which collectively contribute to its robust antioxidant functionality. Chlorogenic acid interacts with key antioxidant enzymes, such as xanthine oxidase, inhibiting its activity and reducing the production of oxygen free radicals in the body [53]. According to Wang et al. [54], computational molecular docking analysis revealed that chlorogenic acid binds to the FAD region of xanthine oxidase, leading to its inhibition. The active form of xanthine oxidase is a homodimer, and each subunit contains one molybdopterin cofactor, two distinct [2Fe-2S] centers, and one flavin adenine dinucleotide (FAD) cofactor that shuttles electrons away from the active site following xanthine or hypoxanthine oxidation [55]. FAD serves as the cofactor for numerous proteins with diverse functions, such as electron transport, redox catalysis, and oxygen activation [56]. Docking simulations indicated that chlorogenic acid, which bears a bulky hydrophilic quinyl group, binds more strongly to the FAD region (docking energy of −12.40 kcal/mol) compared to the molybdopterin region (docking energy of −7.58 kcal/mol). This stronger binding likely hampers oxygen’s approach to the site, thereby impeding electron transfer from the molybdopterin center and arresting the oxidation process [54].

The antioxidant properties of chlorogenic acid are exerted through multiple mechanisms. The phenolic hydroxyl group within its structure exhibits a high degree of reactivity with free radicals, facilitating the formation of hydrogen radicals. These hydrogen radicals possess an antioxidant effect, effectively neutralizing the activity of hydroxyl radicals and superoxide anions. Additionally, chlorogenic acid inhibits lipid peroxidation by modulating the activity of antioxidant enzymes, enhancing amino acid and glutathione metabolism, and promoting lipid metabolism [57]. A study using molecular docking and spectroscopy demonstrated that chlorogenic acid inhibits lipid oxidation by directly interacting with key enzymes such as lipoxygenase and endogenous lipase. Docking simulations suggest that chlorogenic acid binds near the active site of lipase through hydrogen bonds, hydrophobic interactions, and Van der Waals forces, altering its conformation and activity. These structural modifications, confirmed by fluorescence and spectral analyses, contribute to chlorogenic acid’s inhibitory effects on lipid peroxidation, reinforcing its potential as a natural antioxidant [58].

Beyond its direct antioxidant activity, chlorogenic acid also plays a crucial role in gene expression regulation and cellular signaling. It activates the Nrf2/ARE signaling pathway by inhibiting the ubiquitin-mediated degradation of the Nrf2 protein, stabilizing its cytoplasmic concentration, and augmenting its transcriptional activity under stress conditions [59]. The molecular interaction between Keap1 and Nrf2 has been demonstrated to facilitate the ubiquitin-mediated proteasomal degradation of Nrf2, thereby modulating its biological function. In addition, molecular docking analyses revealed structural and energetic insights into chlorogenic acid’s interaction with Keap1’s kelch domain. The study revealed a binding energy of −9.1 kJ/mol and three hydrogen bond interactions, suggesting a favorable interaction between the two molecules. Chlorogenic acid has been identified as a potential Nrf2 signaling activator, operating through the downregulation of its Keap1 repressor [60]. Furthermore, chlorogenic acid has been shown to exert a positive regulatory effect on the PI3K-Akt signaling pathway, a critical mediator of cellular apoptosis and a key player in the MAPK signaling pathway. This regulatory relationship further reinforces the antioxidant effects of chlorogenic acid [57]. Bio-informational analysis revealed that chlorogenic acid targeted key genes (AKT, MAPK1, MAPK14, NF-κB, TNF, IL-2, and IL1B) by regulating the PI3K/Akt and NF-κB/MAPK signal pathways [61].

The antioxidant properties of flavonoids are known to increase with the presence of a hydroxyl group at position 3 of the heterocyclic ring C. This effect is further enhanced by the combination of hydroxyl groups at positions 3′ and 4′ of ring B, forming a conjugated system with the double bond between carbons 2 and 3 of ring C. Additionally, the presence of a 4-oxo functional group on ring C contributes significantly to the antioxidant properties of flavonoids [53]. Catechins possess phenolic hydroxyl groups at positions 3, 5, and 7 on ring A and positions 3′ and 4′ on ring B, which stabilize free radicals, enabling both direct and indirect antioxidant activities.

Catechins act as free radical scavengers by donating an electron, thereby stabilizing the resulting radicals through resonance. In addition to this direct action, catechins have been shown to regulate protein synthesis, signaling pathways, and antioxidant enzyme production while inhibiting pro-oxidant enzymes such as NADPH oxidase [62,63]. Molecular docking studies predict that catechins interact with multiple NADPH oxidase subunits, with varying binding affinities. The strongest predicted binding affinities were observed for p40phox and p67phox PB1 subunits, with binding energies ranging from −8.3 to −9.9 kcal/mol. In contrast, a comparatively weaker affinity was predicted between the catechin compounds and the gp91 (phox) subunit, with binding energies ranging from −4.9 to −6.5 kcal/mol. In summary, the study predicted that catechin compounds possess drug-likeness properties and have affinities for interaction with NADPH oxidase subunits, particularly p40phox and p67phox PB1, which is likely to exert their antioxidant effects [64]. Catechin has also been identified as a potential Nrf2 signaling activator, operating through the downregulation of its Keap1 repressor. Molecular docking studies of catechin relative to the Keap1 kelch domain showed that they have −9.2 kJ/mol binding energy with four hydrogen bond interactions [60]. Furthermore, catechins have been observed to modulate oxidative stress-related pathways, thereby reducing inflammation by affecting redox-sensitive transcription factors such as NF-κB and AP-1 [63]. These compounds have been shown to interact with cell membranes and proteins via their hydrophobic benzene rings and hydrogen bonding, and their structural similarity to ATP allows for competitive binding to enzyme ATP-binding sites, influencing various cellular functions [62].

**Figure 1:**
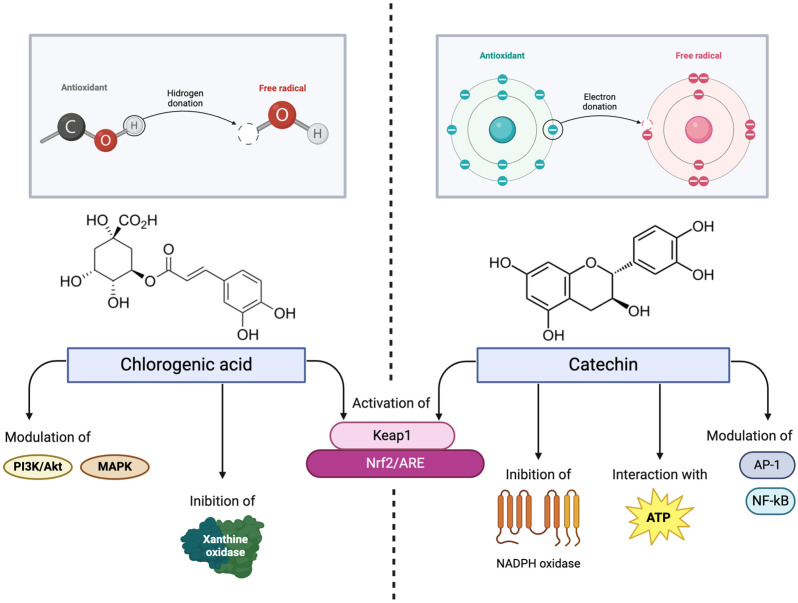
Comparison of the antioxidant mechanisms of chlorogenic acid and catechins. Chlorogenic acid scavenges radicals via hydrogen donation, inhibits xanthine oxidase, and activates the Nrf2/ARE pathway through Keap1 interaction. Catechins neutralize radicals via electron donation, inhibit NADPH oxidase, and modulate NF-κB and AP-1. Both compounds enhance Nrf2 signaling, upregulating antioxidant genes for cellular protection.

Overall, phenolic compounds, such as chlorogenic acid and catechins, demonstrate potent antioxidant effects through free radical scavenging, the modulation of oxidative stress pathways, and enzymatic regulation. Their structural features, including hydroxyl group positions and conjugated systems, enhance their efficacy, enabling them to reduce inflammation, protect cells, and support overall oxidative balance (Figure 1). These properties highlight their potential for pharmaceutical applications in managing oxidative stress-related conditions.

## 5. Antibacterial Properties of Phenolic Compounds from *P. communis*

In addition to antioxidant properties, phenolic compounds can contribute to antibacterial activity [65]. The main pear cultivars have been explored in terms of antioxidant activity. This review article, therefore, offers novel insights for researchers engaged in this field. Current understanding is primarily based on in vitro research, so its applicability in clinical environments remains uncertain. A limitation of the use of phenolic compounds derived from plants as antibacterials is the lack of standardized extraction methods [66]. Parameters like the time of extraction, temperature, ratio of solvent/sample, number of extractions, and type of solvent can all affect phenolic extraction [67]. The antibacterial effects of phenolic extracts derived from *P. communis* are not extensively documented; however, three studies have reported antibacterial properties among these compounds [17,18,19]. Therefore, this review compiled the extraction conditions and antibacterial impacts of these four studies (Table 4).

Sroka et al. [17] investigated the antibacterial activity of four dry residues extracted from phenolic compounds in Conference pear leaves. Methanol extraction and subsequent fractionation produced four residues: EA (methanol extract), EB (water extract), EC (ethyl acetate extract), and ED (residue obtained from an aqueous solution). The extract’s antibacterial properties were evaluated using both Gram-positive strains (namely, *Staphylococcus aureus* ATCC 25923, methicillin-resistant *S. aureus* K326, *Bacillus subtilis* ATCC 6633, *Enterococcus faecalis* ATCC 25212, and *Helicobacter pylori* J99) and Gram-negative strains (namely, extended-spectrum β-lactamase *Escherichia coli* 295, *E. coli* ATCC 29522, and *Pseudomonas aeruginosa* ATCC 27853). The EC extract exhibited the strongest antibacterial effects, while ED had the weakest. *H. pylori* was the most inhibited bacterium, while ESBL-*E. coli*, known for its high resistance, showed sensitivity to EC extracts. The results showed that the EC extract, which demonstrated the strongest antibacterial activity, also had the highest hydroquinone content (131.3–272.8 mg/g DW). A strong correlation between hydroquinone concentration and antibacterial activity was evident, suggesting that this compound plays a central role in bacterial inhibition. In contrast, the ED extract, which had the weakest activity, contained the lowest hydroquinone concentration (0.24–0.48 mg/g DW). These findings reinforce the relationship between hydroquinone levels in the extracts and their antibacterial efficacy. However, the study did not report minimum inhibitory concentration (MIC) values, which would have further quantified the antibacterial potency of hydroquinone and other phenolic compounds. Hydroquinone outperformed other phenolics like quercetin, caffeic acid, and tannin in antibacterial potency, as reported by Żbikowska et al. [69]. Its mechanisms, including damage to bacterial cell walls and increased membrane permeability, were demonstrated by Ma et al. [70]. Sroka et al. [17] further confirmed a high correlation between antibacterial effects and total phenolic content in the extracts, highlighting hydroquinone as the primary antibacterial agent.

Zbigniew et al. [18] evaluated *P. communis* leaf extracts for antibacterial activity and phenolic content, focusing on hydroquinone and arbutin. This study evaluated the ability of four extracts, namely WA (20% methanol), WB (water), WC (ethyl acetate), and WD (methanol), to inhibit the growth of Gram-positive (*S. aureus* ATCC 25923, methicillin-resistant *S. aureus* (clinical strain), and *B. subtilis* ATCC 6633), and Gram-negative bacteria (*H. pylori* J99, *E. coli* ATCC 25922, ESBL-*E. coli* (clinical strain), and *P. aeruginosa* ATCC 27853). The WC extract, which had the highest hydroquinone levels (0.073 mg/g), showed the strongest antibacterial effects. *H. pylori* was the most sensitive bacterium, while *E. coli* and *P. aeruginosa* exhibited low sensitivity and ESBL-*E. coli* was completely resistant. Statistical analysis revealed a strong positive correlation between hydroquinone concentration and antibacterial activity but no significant link between antibacterial effects and total phenolic content, differing from Sroka et al. [17]. However, the study did not report MIC values, limiting the ability to quantitatively compare the antibacterial potency of the extracts. *E. coli* and *P. aeruginosa* displayed considerably lower sensitivity, whereas ESBL-*E. coli* demonstrated complete insensitivity. Overall, the ethyl acetate extract, which boasted substantially higher hydroquinone levels than its counterparts, emerged as the most effective. In addition, the statistical analysis performed by Zbigniew et al. [18] (Student’s *t*-test) confirmed a positive association concerning the antibacterial activity of these extracts and their hydroquinone concentration. While Sroka et al. [17] found a strong correlation between antibacterial effects and total phenolic content, Zbigniew et al. [18] observed this correlation only with hydroquinone, suggesting that hydroquinone may be the key factor in the antibacterial activity of *P. communis* leaf extracts in their study.

Erbil et al. [19] examined the antibacterial properties of fruit from five *P. communis* varieties (Deveci, Kizil, Egirsah, Gugum, and Banda). In contrast to the two previously mentioned studies, this study investigated the antibacterial effects of water pear fruit extracts through the agar well diffusion technique. The bacteria tested also included Gram-positive (*S. aureus* ATCC 6538, *B. subtilis*, *Bacillus licheniformis*, and *Bacillus megaterium* DSM 32) and Gram-negative strains (*Enterobacter aerogenes*, *Klebsiella pneumoniae*, *E. coli*, and *P. aeruginosa* ATCC 9027). The extract from the Banda pear exhibited the highest level of bacterial inhibition against *P. aeruginosa*, with an inhibition halo of 20.140 mm. Conversely, the extract from the Gugum pear showed the lowest level of antibacterial activity against *E. coli*, with an inhibition halo of 12.343 mm. Additionally, no pear cultivar extracts showed antibacterial activity against *B. licheniformis*. According to the authors, this is the first report on the antibacterial properties of the leaves of the pear varieties included in this work.

Ferreira et al. [68] evaluated how particle size and extraction methods affect the phenolic content and antibacterial activity of pear pomace powder with varying mesh sizes (1 mm, 710 μm, 180 μm, 75 μm, and 53 μm). Two extraction methods were employed: one using 80% methanol with ultrasonic treatment and shaking, and the other using hexane with the Soxhlet method. The antibacterial activity of each sample was quantified using the microdilution method against *S. aureus* ATCC 6538 and *E. coli* O157:H7 NCTC 12900. The results demonstrated a discernible inhibitory effect on *E. coli* relative to *S. aureus*. This was attributed to the fact that *S. aureus* is a species that is frequently resistant to antibiotics. Bacterial growth was inhibited for both strains but more evidently in the two-phase extraction samples. These results corroborate the hypothesis that processing pear pomace could be a viable approach to enhancing the antibacterial activity of bacteria.

Both Erbil et al. [19] and Ferreira et al. [68] did not investigate individual phenolic compounds or assess MIC values, which limits understanding of the specific mechanisms behind the antibacterial activity.

Güven et al. [71] explored the antibacterial properties of *P. communis* subsp. *communis* pulp using the agar well diffusion method. Fresh fruit samples were subjected to Soxhlet extraction with ethyl acetate. Various microorganisms including human and plant pathogens, food contaminants, and saprophytes were tested, and the extracts of *P. communis* subsp. *communis* suppressed the development of several of these microorganisms. To our understanding, this study is the primary demonstration of the antibacterial properties of ethyl acetate extracts in *P. communis* subsp. *communis* fruit.

The antibacterial efficacy of *P. communis* extracts varied depending on the solvent used for extraction. Ethyl acetate extracts demonstrated the strongest antibacterial activity, particularly against *H. pylori* and ESBL-*E. coli* [17,18]. Methanol and water extracts exhibited comparatively weaker antibacterial effects. In studies on pear fruit extracts, water-based extracts showed significant inhibition against *P. aeruginosa*, particularly in the Banda pear variety, whereas the Gugum pear extract displayed the lowest inhibition against *E. coli* [19]. Additionally, the ultrasonic-assisted methanol extraction of pear pomace demonstrated inhibitory effects against *S. aureus* and *E. coli* [68].

Table 5 presents a comparison of the antibacterial efficacy of ethyl acetate used to extract phenolic compounds from *P. communis* against a range of microorganisms. The table includes the inhibition zones caused by ethyl acetate, alongside the inhibition zones induced by standard antibiotics for each microorganism tested. The antibiotic susceptibility data were sourced from CLSI guidelines, offering a reference for evaluating the relative potency of the extracts in comparison to commonly used treatments [72].

The ethyl acetate extracts of *P. communis* show promising antibacterial activity, particularly against *S. aureus* and *E. faecalis*, with inhibition zones comparable to or even greater than those of some antibiotics, such as ciprofloxacin (21 mm) and chloramphenicol (18 mm). While the extracts exhibit moderate activity against *E. coli* and *P. aeruginosa*, they are generally less effective than the antibiotics tested, particularly against *P. aeruginosa*. Ciprofloxacin, for example, shows consistently strong inhibition zones across all microorganisms, highlighting its superior efficacy. Overall, while pear extracts demonstrate significant antibacterial potential, further research is needed to confirm their efficacy, especially against Gram-negative bacteria, and explore their potential as alternatives or adjuncts to conventional antibiotics.

### Molecular Mechanisms of Antibacterial Action

**Figure 2:**
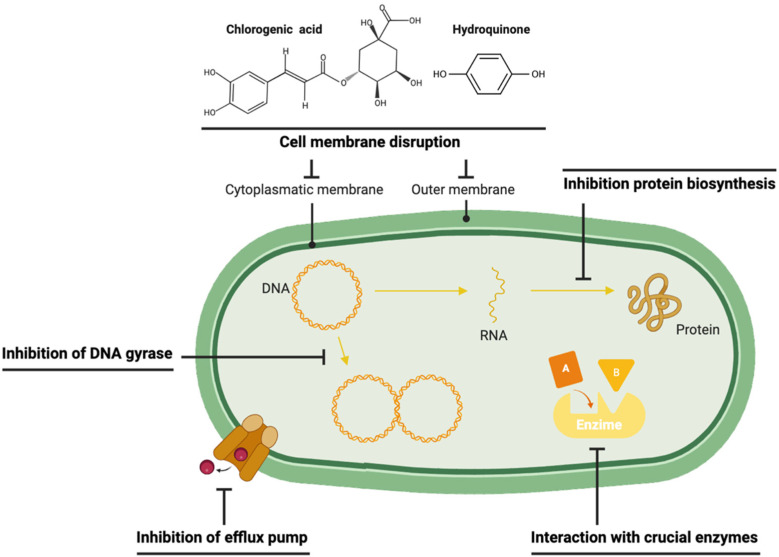
Different molecular mechanisms of action of phenolic compounds.

Phenolic compounds are recognized to act as antibacterial agents by penetrating or disrupting membranes (Figure 2) [73]. Polyphenols bind with membranes through multiple locations, allowing them to act more effectively as antibacterial agents [74]. Their antibacterial mechanisms of action are typically more efficient against Gram-positive bacteria, possibly due to the presence of an outer membrane of a phospholipid/lipopolysaccharide bilayer in Gram-negative bacteria, which acts as an obstacle to conjugation with phenolic compounds [73]. Different phenolic compounds may act together to increase or decrease their antibacterial efficacy against pathogenic bacteria, so it is essential to study not only the antibacterial activity of isolated elements but also the whole byproduct [75].

Hydroquinone is recognized as the key to the antibacterial effects of pear leaves by Sroka et al. [17] and Zbigniew et al. [18]; however, other phenolic compounds with antibacterial properties have also been discovered. Arbutin, for instance, is an essential phenolic compound with antibiotic properties that is frequently used to treat urinary tract infections [32]. This phenolic compound, when absorbed in the gastrointestinal tract, is split into aglycone hydroquinone and glucose by the gut flora and under the control of the enzyme β-glucosidase [76]. The absence of β-glucosidase activity is correlated with low antibacterial properties, so it is possible that the antibacterial outcome of arbutin depends on the extracellular action of this enzyme [77,78]. The MIC values of arbutin and hydroquinone against multiple bacteria were estimated by Jurica et al. [77], and arbutin did not display any antibacterial effect, while hydroquinone presented strong antibacterial effects against *E. faecalis*. Likewise, Ma et al. [70] revealed that the MIC values of arbutin against *S. aureus*, MRSA, and ESBL-*S. aureus* were weaker than that of hydroquinone. The authors also noted that both hydroquinone and arbutin were ineffective against Gram-negative bacteria, possibly due to differences in cell wall structures between Gram-positive and Gram-negative bacteria. The outer membrane of Gram-negative bacterial cell walls consists of lipoproteins, lipid bilayers, and lipopolysaccharides. The lipid bilayer is like the cell membrane [79]. In addition, Gram-negative bacteria cell walls contain a periplasmic space, which contains a variety of enzymes, including protease, nuclease, and detoxification enzymes. These enzymes play an important role in bacterial antidrug-resistant activity [70]. These factors may cause hydroquinone and arbutin to be ineffective against Gram-negative bacteria.

Chlorogenic acid demonstrated bactericidal properties against various bacterial strains, including trimethoprim/sulfamethoxazole-resistant *Stenotrophomonas maltophilia* [80], *Yersinia enterocolitica* [81], *K. pneumoniae* [16], *P. aeruginosa* [82], *H. pylori*, *E. coli*, *Salmonella enteritidis*, and *Pseudomonas fluorescens* [83]. The mechanisms by which chlorogenic acid exerts its antibacterial effects involve the disruption of the permeability of the bacterial cell membrane, leading to the efflux of intracellular constituents, the resultant loss of function, and, ultimately, cell death [82,84]. Consistent with this hypothesis, chlorogenic acid has been shown to improve the permeability of the outer and plasma membranes of *Shigella dysenteriae* and the plasma membrane of *Streptococcus pneumoniae*, resulting in the leakage of cytoplasmic contents, including nucleotides [84]. Mujtaba et al. [83] suggested that chlorogenic acid induced the leakage of intracellular proteins and ATP from *P. aeruginosa*. Chen et al. [81] stated that chlorogenic acid can cause harm to the cell wall and cell membrane of *Y. enterocolitica.* Furthermore, Karunanidhi et al. [80] have characterized chlorogenic acid as a promising in vitro antibacterial and antibiofilm agent for the management of *Stenotrophomonas maltophilia.*

## 6. Therapeutic Potential of *P. communis* Phenolic Compounds

The interest in *P. communis*-derived phenolic compounds is attributable to their potential applications in the medical and pharmacological domains, particularly in the context of combating infections caused by antibiotic-resistant pathogens. This section explores the medical and pharmaceutical applications of these compounds, their synergy with antibiotics, and the necessity for in vivo studies to validate their therapeutic efficacy.

### 6.1. Medical and Pharmaceutical Applications

Phlorizin, identified in the peel, pulp, and seed of different *P. communis* cultivars, is currently sourced from apple pomace for its antibacterial properties [34,85]. Additionally, this flavonoid exhibits various pharmacological effects [86,87]. Extracts rich in anthocyanins and catechins have demonstrated inhibitory effects on foodborne pathogens and contribute to intestinal antibacterial defense. In vivo studies indicate that consuming these compounds can influence foodborne illness outcomes by modulating gut microbiota [88]. Moreover, quercetin has shown antibacterial and antibiofilm activity against *S. aureus* and *S. saprophyticus* [89].

Recent studies suggest that polyphenols derived from *P. communis* possess antiproliferative properties against bladder cancer cells, indicating potential therapeutic applications in oncology [44]. Furthermore, the *P. communis* peel, which is particularly rich in phenolic antioxidants, is a promising candidate for anti-aging and skin-protective formulations. While pear pomace extracts have been explored in cosmetics, challenges related to stability remain a limitation [27,68].

Beyond their antioxidant capacity, *P. communis* phenolics exhibit potent antibacterial activity, positioning them as viable alternatives to synthetic antibacterials. Hydroquinone and chlorogenic acid have demonstrated the inhibition of various bacterial pathogens, with effects comparable to conventional antibacterial agents, suggesting potential applications in hospital disinfectants or as therapeutic adjuvants [17,80]. However, while chlorogenic acid is already utilized in cosmetics and food preservation, hydroquinone’s therapeutic application remains limited due to its toxicity, including potential carcinogenic effects [90,91,92]. As concerns grow regarding the safety of synthetic preservatives like triclosan and parabens, natural phenolic compounds offer a more sustainable and biodegradable alternative [93].

These phenolics also show promise in antibacterial formulations for wound healing and skin infections given their anti-inflammatory properties and ability to promote tissue regeneration, potentially reducing conventional antibiotics [94,95]. Additionally, arbutin—a glycoside of hydroquinone present in *P. communis*—is widely utilized for urinary tract infection (UTI) prevention due to its conversion into hydroquinone in the urinary tract, where it exhibits antibacterial activity against uropathogenic bacteria [70,77]. Furthermore, phenolic compounds such as catechins and quercetin support immune support function and gut health by promoting beneficial microbial populations while inhibiting pathogenic bacteria, reinforcing their potential as natural prebiotics [96,97]. Developing more environmentally friendly extraction methods and stable formulations could enable these phenolics to replace synthetic antibacterials, reducing environmental impact while providing effective natural alternatives in medicine and pharmaceuticals.

### 6.2. Synergy with Antibiotics

The synergistic effect between phenolic compounds and antibiotics represents a promising strategy to restore the efficacy of antibacterial agents against resistant bacteria [98]. Studies have demonstrated that phenolics can enhance antibiotic activity through multiple mechanisms, including increasing bacterial membrane permeability, which facilitates antibiotic penetration and reduces the MIC values. Additionally, certain phenolics act as efflux pump inhibitors, preventing bacteria from expelling antibiotics and thereby enhancing their intracellular retention [99]. Hydroquinone and flavonoids such as quercetin have been identified as potential modulators of these resistance mechanisms, while chlorogenic acid has shown synergy with β-lactam antibiotics against carbapenem-resistant *K. pneumoniae* [70,100]. Similarly, catechin has demonstrated synergistic action when in combination with norfloxacin and gentamicin against *S. aureus*, as well as additive effects when combined with aminoglycosides against *P. aeruginosa* [101,102]. Moreover, quercetin has been reported to enhance the susceptibility of *S. aureus* to ciprofloxacin and gentamicin by disrupting its cell membrane integrity and increasing reactive oxygen species production, leading to enhanced bacterial cell lysis [103]. These findings suggest that *P. communis* phenolic extracts could be incorporated into pharmaceutical formulations to enhance the effectiveness of conventional antibiotics, allowing for lower doses, minimizing side effects, and potentially mitigating the development of antibiotic resistance.

### 6.3. In Vivo Studies on Therapeutic Efficacy

While multiple in vitro studies have confirmed the antibacterial and antioxidant activity of *P. communis* phenolic compounds, further validation through in vivo models and clinical trials is essential to determine their therapeutic efficacy. A key aspect to consider is the bioavailability and metabolism of these compounds, as chlorogenic acid and catechin undergo intestinal and hepatic biotransformation, potentially generating active metabolites with distinct pharmacological properties that could enhance or modify their therapeutic effects [104,105].

Animal infection models can provide valuable insights by comparing the antibacterial potential of *P. communis* phenolics with conventional antibiotics, helping to elucidate their systemic effects and optimal therapeutic applications. Notably, hydroquinone has demonstrated in vivo antibacterial activity in urinary tract and skin infections by blocking *E. coli* adhesion to the urinary epithelium and preventing bacterial invasion and biofilm formation, highlighting its potential for therapeutic use [106].

Clinical trials are crucial for evaluating the safety and efficacy of phenolic-based pharmaceutical formulations, particularly in the treatment of infections, immune modulation, and gut health. Initial studies could explore their role as adjuncts to antibiotic therapy, targeting multidrug-resistant bacterial infections while also assessing their impact on microbiota modulation. Advancing in vivo research will be key to optimizing extraction techniques, improving formulation stability, and establishing *P. communis* phenolics as viable candidates for pharmaceutical and medical applications.

## 7. Challenges and Future Perspectives

Despite the promising potential of *P. communis* phenolic compounds, there are several challenges that must be addressed to ensure their effective application. A significant obstacle is the lack of standardized extraction methods, leading to variability in composition and bioactivity. Additionally, bioavailability remains a major concern, requiring further research to understand the absorption, metabolism, and stability of these compounds in the human body.

Another major limitation lies in the isolation and scalability of phenolic compounds from pear residues. The complexity of plant matrices, where multiple phenolics coexist in varying concentrations, makes it difficult to obtain pure active compounds in high yields. Additionally, some phenolic compounds may degrade or lose bioactivity during purification processes, which can impact their therapeutic potential. Cost-effective and sustainable large-scale extraction methods must be developed to support commercial viability. While ethyl acetate has shown promise as a solvent for extracting antibacterial compounds, further studies are needed to compare its efficiency with conventional antibiotics.

A critical challenge limiting pharmaceutical applications is cytotoxicity. Some *P. communis* phenolics, particularly hydroquinone, have been linked to nephrotoxicity, genotoxicity, and carcinogenic potential [107]. Hydroquinone induces oxidative stress, leading to DNA damage and apoptosis, prompting regulatory restrictions from agencies such as the FDA and EMA [108,109]. In contrast, its glycosylated derivative, arbutin, demonstrates antibacterial activity with reduced toxicity due to its controlled hydrolysis [76,110]. However, long-term safety assessments of arbutin and other phenolic derivatives are still needed. Future research should explore strategies to mitigate hydroquinone toxicity, including structural modifications, encapsulation, and targeted delivery systems.

Beyond cytotoxicity, stability remains a challenge, particularly in cosmetic applications. Phenolics exhibit strong antioxidant properties, but maintaining their bioactivity in formulations over time has proven difficult. Advances in encapsulation and stabilization techniques could expand their use in dermatological products [27,68].

To enhance clinical applicability, further comparative studies with synthetic antibiotics are necessary, particularly regarding MIC values and direct antibacterial efficacy. Additionally, the potential activity of *P. communis* phenolics against fungal and viral pathogens remains underexplored and could provide new therapeutic opportunities.

Regulatory approval is another key hurdle. The transition from in vitro studies to pharmaceutical applications requires rigorous toxicological and clinical validation to meet FDA and EU standards. Future research should prioritize in vivo efficacy studies, pharmacokinetic assessments, and clinical trials to optimize their formulation for medical use.

Addressing these challenges requires a multifaceted approach, including the following: developing standardized and sustainable extraction methods, optimizing formulation stability for pharmaceutical and cosmetic applications, investigating bioavailability and metabolic stability to enhance therapeutic efficacy, exploring the synergistic effects of *P. communis* phenolics with synthetic antibiotics and antioxidants, expanding research to include fungal and viral pathogens, and incorporating antibiotic controls in future studies to facilitate direct comparisons. By tackling these issues, *P. communis* phenolic compounds could be effectively integrated into food, pharmaceutical, and cosmetic applications, maximizing their potential benefits.

## 8. Conclusions

This review highlights the phenolic profile of *P. communis* residues and their potential as bioactive compounds with antioxidant and antibacterial activities. Chlorogenic acid and catechin were identified as key contributors to antioxidant activity, while hydroquinone and chlorogenic acid exhibited strong antibacterial activity, particularly when extracted using ethyl acetate. These phenolic compounds demonstrate promising mechanisms of antibacterial action, including bacterial membrane disruption, enzyme inhibition, and the modulation of oxidative stress, making them valuable candidates for combating antibacterial resistance.

Beyond their potential in food preservation, pharmaceuticals, and cosmetics, *P. communis*-derived phenolics emerge as sustainable alternatives to synthetic antibacterials, addressing growing concerns about bacterial resistance and environmental impact. However, despite their promising biological activities, key challenges remain, including low bioavailability, the need for extraction standardization, and scalability for industrial applications. Future research should prioritize in vivo efficacy studies, pharmacokinetic assessments, and clinical trials to validate their therapeutic potential and optimize their formulation for medical use. Additionally, exploring synergistic interactions between phenolics and conventional antibiotics could pave the way for innovative combinatorial therapies against multidrug-resistant pathogens.

Using *P. communis* byproducts not only enhances sustainability but also adds value to the agro-industrial sector, fostering the development of eco-friendly, multifunctional bioactive agents. With further advancements in extraction techniques and therapeutic validation, these phenolic compounds could play a crucial role in future natural antibacterial strategies for food, pharmaceutical, and medical applications.

## Figures and Tables

**Table 1:** Extraction conditions and yield of phenolic compounds from *P. communis*.

Matrices	Sample Weight	Solvent	Time	Yield of Phenolic Compounds (g GAE kg^−1^)	References
Peel	5 g	10 mL methanol/formic acid/BHT (96:3:1% *m*/*v*)	1 h	0.2–1.1	[15]
Pulp	10 g			0.008–0.08	
Peel	0.2 g	5 mL methanol/ascorbic acid (99:1% *m*/*v*)	20 min	91.8	[30]
Pulp				23.4	
Seeds				97.9	
Leaves				532.7	
Peel	10 g	100 mL methanol/water (60:40 *v*/*v*) (three times)	30 min (each time)	26.4–112.2	[31]
Pulp				2.5–34.8	
Peduncle	250 mg	3 mL methanol/water (80:20 *v*/*v*)	24 h	42.2–58.8	[32]
Exocarp				16.5–40.3	
Mesocarp				6.4–17.7	
Endocarp				18.3–40.4	
Peel	10 g	100 mL methanol/hydrochloric acid (99.9:0.1 *v*/*v*)	1 h	116.3–173.1	[33]
Pulp				85.3–146.4	
Peel	2 g	20 mL methanol/water/formic acid (19.95:49.95:0.10 *v*/*v*/*v*)	25 min	0.7–1.1	[34]
Pulp				0.2–0.3	
Seeds				0.5–1.4	
Pulp	5 g	20 mL ethanol (70:30 *v*/*v*)	12 h	0.2–0.3	[35]
Peel	40 g	200 mL water	72 h	3.3–4.0	[19]
Pulp				1.3–2.1	

**Table 2:** Main phenolic compounds in distinct anatomical elements of *P. communis*.

Phenolic Compounds	Leaf	Peduncle	Peel	Pulp	Core	Seed	References
Flavonols							
Isorhamnetin	+	+		+		+	[29,32]
Kaempferol	+		+	+		+	[29,30,33,35]
Quercetin	+	+	+	+		+	[15,29,31,32,34,35]
Flavanols							
Catechin	+	+	+	+	+	+	[15,29,31,32,34,35]
Epicatechin	+	+	+	+	+	+	[15,29,31,32,34,35]
Procyanidin	+		+	+		+	[29,33,35]
Anthocyanins							
Cyanidin			+	+			[29,30,35]
Hydroxycinnamic acids							
Caffeic acid	+		+	+		+	[30,34,35]
Chlorogenic acid	+	+	+	+	+	+	[15,29,31,32,33,34]
Ferulic acid	+		+	+		+	[29,30,31,34,35]
p-Coumaric acid			+	+		+	[29,31,34]
Hydroxybenzoic acids							
Gallic acid		+	+	+		+	[31,32,34,35]
Hydroxybenzoic acid			+	+		+	[34,35]
Vanillic acid			+	+		+	[31,34]
Phenolic glycosides							
Arbutin	+	+	+	+	+	+	[15,29,31,32,35]

**Table 3:** An overview of the phenolic compound matrices of the three most produced European pear cultivars that have been exploited in terms of antioxidant activity over the past decade.

Pear Cultivars	Matrices	Antioxidant Capacity Assays		References
Conference	Pulp	DPPH	238.1 ± 12.1 (EC50, µg FW)	[42]
		ABTS	128.4 ± 7.3 (µmol TE/100 g FW)	
		FRAP	174.6 ± 8.8 (µmol TE/100 g FW)	
	Fruit ^a^	FRAP	227.6 ± 7.6 (µmol TE/100 g FW) ^c^	[43]
			193.0 ± 30.1 (µmol TE/100 g FW) ^d^	
	Fruit	DPPH	398.0 ± 10.0 (µmol TE/100 g DW)	[44]
		FRAP	437.0 ± 11.0 (µmol TE/100 g DW)	
	Edible portion	DPPH	16.5 ± 0.8 (µmol TE/100 g FW)	[45]
		ABTS	130.0 ± 4.2 (µmol TE/100 g FW)	
		FRAP	10.4 ± 0.2 (µmol TE/100 g FW)	
	Pear Slices ^b^	DPPH	395.3 ± 0.4 (µmol TE/100 g DW) ^e^	[46]
		ABTS	775.8 ± 1.3 (µmol TE/100 g DW) ^e^	
		FRAP	278.3 ± 0.1 (µmol TE/100 g DW) ^e^	
William BC	Pulp	DPPH	163.2 ± 0.1 (µmol TE/100 g FW) ^e^	[47]
		ABTS	242.9 ± 7.4 (µmol TE/100 g FW) ^e^	
		FRAP	214.6 ± 1.9 (µmol TE/100 g FW) ^e^	
	Fresh fruit	DPPH	33.0 ± 1.2 (%inhibition)	[3]
	Dried fruit		66.6 ± 2.2 (%inhibition)	
	Fruit ^a^	FRAP	170.6 ± 20.5 (µmol TE/100 g FW) ^c^	[43]
			142.2 ± 8.1 (µmol TE/100 g FW) ^d^	
	Leaf	DPPH	280.0 ± 1.0 (µmol TE/100 g DW)	[48]
Rocha	Pulp	ABTS	2025.1 ± 292.9 (µmol TE/100 g FW) ^f^	[49]
	Fresh fruit	DPPH	9307.5 ± 872.6 (EC50, µg FW) ^e^	[50]
	Pomace	DPPH	606.6 ± 138.8 (µmol TE/100 g DW) ^e^	[51]
		FRAP	90.2 ± 3.2 (µmol TE/100 g DW) ^e^	
		DPPH	362.0 ± 1.2 (µmol TE/100 g FW)	[6]
		FRAP	892.0 ± 49.8 (µmol TE/100 g FW)	
	Puree	DPPH	306.0 ± 31.7 (µmol TE/100 g FW)	
		FRAP	730.0 ± 30.9 (µmol TE/100 g FW)	
	Peel	ABTS	6877.1 ± 1298.5 (µmol TE/100 g FW) ^f^	[49]

^a^ Seeds and core were removed; ^b^ seeds and stalks were removed; DPPH—2,2-diphenyl-1-picrylhydrazyl; ABTS—2,2′-azinobi-3-ethylbenzothiazoline-6-sulfonic acid; FRAP—Ferric Reducing Antioxidant Power; TE—Trolox Equivalents; FW—Fresh Weight; ^c^ mean values from 2014; ^d^ mean values from 2015; DW—Dry Weight; ^e^ values extracted from graphical data using PlotDigitizer™ (https://plotdigitizer.com/app accessed on 22 February 2025), slight deviations may exist; ^f^ mean values from five different locations; data are expressed as mean ± SD.

**Table 4:** Antibacterial effects of phenolic compounds extracted from *P. communis*.

Matrices	Method	Cultivar	Extract	Gram-Positive Bacteria						Gram-Negative Bacteria						Reference
				*S. aureus*	MRSA	*B. subtilis*	*E. faecalis*	*B. licheniformis*	*B. megaterium*	*E. coli*	ESBL *E. coli*	*P. aeruginosa*	*H. pylori*	*E. aerogenes*	*K. pneumoniae*	
				Antibacterial Effects (mm)												
Leaf	Disc diffusion	Conference	EA	22 ^a^	23 ^a^	13 ^a^	12 ^a^	-	-	10 ^a^	7 ^a^	14 ^a^	22 ^a^	-	-	[17]
			EB	15 ^a^	14 ^a^	3 ^a^	3 ^a^	-	-	0 ^a^	0 ^a^	3 ^a^	18 ^a^	-	-	
			EC	33 ^a^	36 ^a^	13 ^a^	22 ^a^	-	-	17 ^a^	16 ^a^	22 ^a^	48 ^a^	-	-	
			ED	11 ^a^	14 ^a^	0 ^a^	0 ^a^	-	-	0 ^a^	0 ^a^	0 ^a^	0 ^a^	-	-	
		n.m.	WA	13	10	15	-	-	-	0	0	0	16	-	-	[18]
			WB	0	0	0	-	-	-	0	0	0	10	-	-	
			WC	30	20	23	-	-	-	17	0	12	22	-	-	
			WD	0	0	12	-	-	-	0	0	0	7	-	-	
Fruit	Well diffusion	Gugum	n.a.	16	-	14	-	0	15	12	-	14	-	15	13	[19]
		Banda		16	-	18	-	0	18	18	-	20	-	18	17	
		Kizil		16	-	15	-	0	15	15	-	19	-	15	16	
		Deveci		16	-	0	-	0	14	0	-	0	-	13	15	
		Egirsah		16	-	16	-	0	17	15	-	17	-	16	16	
				Antibacterial Effects (% Bacterial Growth)												
Pomace powder	Microdilution	n.m.	M	84.1 ^b^	-	-	-	-	-	78.2 ^b^	-	-	-	-	-	[68]
			TSE	28.7 ^b^	-	-	-	-	-	11.3 ^b^	-	-	-	-	-	

n.m.—not mentioned; EA—methanol; EB—water; EC—ethyl acetate; ED—residue obtained from aqueous solution; WA—20% methanol; WB—water; WC—ethyl acetate; WD—methanol; ^a^ mean values for the three months (May, July, and September) during which the pear leaf samples were collected; n.a.—not applicable; M—maceration; TSE—two-step extraction; ^b^ mean value for the five particle sizes tested (1 mm, 710 μm, 180 μm, 75 μm, and 53 μm); - denotes not tested.

**Table 5:** Comparison of inhibition zones caused by the most promising phenolic extracts with those induced by recommended antibiotics according to CLSI [72].

	Inhibition Zones for Sensitivity (mm)			
	*S. aureus*	*E. faecalis*	*E. coli*	*P. aeruginosa*
Most promising pear extracts				
EC [17]	33	22	17	22
WC [18]	30	-	17	12
Most recommended antibiotics				
Ampicillin (10 µg)	-	17	17	-
Amoxicillin-clavulanate (20/10 µg)	-	-	18	-
Chloramphenicol (30 µg)	18	18	18	-
Ceftazidime (30 µg)	-	-	23	18
Ciprofloxacin (5 µg)	21	21	26	25
Meropenem (10 µg)	-	-	23	19
Tetracycline (30 µg)	19	19	15	-
Vancomycin (30 µg)	-	17	-	-
